# Systematic Analysis of Breed, Methodological, and Geographical Impact on Equine Sperm Progressive Motility

**DOI:** 10.3390/ani11113088

**Published:** 2021-10-29

**Authors:** Jodie Perrett, Imogen Thea Harris, Christy Maddock, Mark Farnworth, Alison Z. Pyatt, Rebecca Nicole Sumner

**Affiliations:** 1 Hartpury University and Hartpury College, Hartpury House, Gloucester GL19 3BE, UK; jodie.perrett@hartpury.ac.uk (J.P.); imogen.harris@hartpury.ac.uk (I.T.H.); christy.maddock@hartpury.ac.uk (C.M.); 2 The Jeanne Marchig International Centre for Animal Welfare Education, The Royal (Dick) School of Veterinary Studies, Midlothian EH25 9RG, UK; v1mfarnw@exseed.ed.ac.uk; 3 International Office, Veterinary Medicines Directorate, Addlestone KT15 3LS, UK; a.pyatt@vmd.gov.uk; 4 School of Veterinary Medicine and Science, University of Nottingham, Sutton Bonington, Loughborough LE12 5RD, UK

**Keywords:** equine, progressive motility, temporal trends, semen analysis, breed, seasonality, geographical location

## Abstract

**Simple Summary:**

Semen quality is an important indicator of reproductive health and fertility. With adverse temporal trends in human semen quality over the past 50 years paralleled in male animals, there is increasing concern for the causes and implications of perturbed male reproductive health. The evaluation of equine progressive motility (PM), a parameter closely associated with fertility, provides information on the fertilising capacity of equine ejaculate and current reproductive health of the equine stallion. Using systematic analysis, recent trends in equine PM were determined from 696 estimates from 280 individual studies. Temporal trends indicate equine PM has not significantly changed between the years 1990 and 2018. Significant breed, methodological, and geographical variations observed in equine PM may considerably influence actual and reported stallion fertility potential. Information on stallion PM meaningfully contributes to the wider literature on semen quality and provides avenues for future stallion fertility research. This systematic analysis presents the wider challenges associated with semen quality assessment, particularly within the equine sector, and provides recommendations to promote consistency across industry and research.

**Abstract:**

Over the past five decades, there has been increasing evidence to indicate global declines in human semen quality. Parallel adverse trends measured in male animals indicate a potential environmental aetiology. This study evaluated the progressive motility (PM) of stallion ejaculate through a systematic review and meta-analysis. A total of 696 estimates of equine PM from 280 studies, which collected semen samples between the years 1990 and 2018, were collated for meta-analysis. The method of motility analysis, breed, season of collection, and geographical location were extracted. Simple linear regression determined temporal trends in stallion PM. Studies using microscopy estimated PM to be significantly greater compared to computer-automated methods (*p* ≤ 0.001). For Arabian breeds, PM was consistently higher than other breeds. Over time, there was a significant decline in PM for studies from Europe (*n* = 267) but a significant increase for studies from North America (*n* = 259). Temporal trends indicate the fertilising capacity of equine ejaculate has remained consistently high in the last three decades. That being so, variations observed suggest methodological, geographical, and individual stallion differences may significantly influence actual and reported stallion fertility potential.

## 1. Introduction

Semen quality is an important indicator of male reproductive health and fertility [1,2]. Over the past five decades, there has been increasing concern over declining semen quality across species [3,4,5,6]. Reduced sperm count is associated with reduced reproductive potential and the development of reproductive disorders, such as cryptorchidism, hypospadias, and testicular cancer [2,7]. The decline in human male fertility has remained controversial since the first meta-analysis reported a global reduction of 50% in mean sperm density [3]. Aggregation of heterogenous studies and absence of systematic approaches to control bias were identified as limiting the validity of semen quality trends [3,8,9,10].

Subsequent reanalysis and independent studies support global temporal declines in semen quality, suggesting increasing concern around the causes and implications of reduced male fertility [6,11,12,13,14,15]. A rigorous meta-analysis of global trends in human semen quality reported a 50–60% decline in sperm count in North America, Europe, Australia and New Zealand in the period from 1973 to 2011 [6]. Geographical variations in semen quality, manifested by stronger declines in Westernised regions, suggests sperm count may reflect environmental and lifestyle influences.

Global adverse trends in male reproductive health have been shown in different species. Between 1988 and 2014, progressive motility (PM) declined by 30% in a population of breeding dogs, whilst incidences of cryptorchidism increased [5]. Temporal changes in semen quality have been reported in the Breton Draught and Anglo-Arab Thoroughbreds in France (1981–1996 and 1985–1995, respectively), as well as the Holstein Dairy bull in the USA (1965–1995) [4,16,17]. Semen volume decreased for both stallions and bulls alike. Sperm concentration was found to have increased for stallions, yet decreased for bulls over their respective study periods [4,16]. Stallion sperm production remained unchanged between 1981 and 1996; as such, the increasing trend for sperm concentration may be linked to its inverse relationship with seminal volume [16]. As seminal volume is androgen-dependent, this raises concerns for the cause of declining stallion seminal volume [16,18].

Information on stallion semen quality trends is limited to Breton Draught and Anglo-Arab Thoroughbred stallions in the late 1900s and may not be considered generalisable to stallion populations in the 21st century. The development of assisted reproductive technologies (ARTs) mitigates the effects of male factor infertility, diminishing evolutionary pressure for fertility in stallions [19,20,21]. Current fertility indexes in the equine sector are influenced by factors extrinsic to the stallion, such as per-cycle conception, pregnancy, and foaling rates [22,23]. There is a need to evaluate global reproductive trends in the breeding stallion to determine the status of stallion fertility. This review initiates an approach to elucidate stallion PM trends during the period 1990 to 2018, and therefore identify factors impacting stallion PM. A meta-analysis assessing equine PM will meaningfully contribute to the wider literature on semen quality trends and provide recommendations for future research to support stallion fertility. This study presents significant methodological, geographical, and breed variations in stallion PM, representing the wider challenges in the equine sector that need to be addressed to understand the considerable variation in semen quality among stallions.

## 2. Materials and Methods

### 2.1. Ethics

Ethical approval for this study was granted by the Hartpury University Ethics Committee (Ethics Application Number: ETHICS2020-21-LR).

### 2.2. Systematic Review

The conduct and reporting of the systematic review adhered to Meta-analysis Of Observational Studies in Epidemiology (MOOSE) guidelines throughout. A comprehensive search of literature was conducted to identify articles that reported data on equine progressive motility. PubMed (access to MEDLINE database; https://pubmed.ncbi.nlm.nih.gov/), PubAg (the U.S Department of Agriculture and National Agricultural Library search engine https://pubag.nal.usda.gov/), and BASE (Bielefield Academic search engine; https://www.base-search.net/) were searched in the period from 13 January 2021 to 10 February 2021. Boolean search phrases were used to improve the specificity and sensitivity of the search: (stallion* OR equine* OR colt* OR horse*) AND (semen AND quality OR sperm* OR insemin* NOT human*) NOT horseradish. The ‘Mendeley reference management system’ was used to manage studies retrieved from the literature search.

#### 2.2.1. Eligibility Criteria

The review considered all studies that reported primary or retrospective data on equine PM, defined by WHO as “sperm moving actively, either in a linear or large circle, regardless of speed” [1]. Studies were included if semen samples were collected from healthy reproductively normal stallions using standard procedures. Microscopy and computer-assisted sperm analysis (CASA) were considered acceptable methods of PM assessment (Table 1). Typically, stallion sperm quality is assessed via assessment of morphology and motility but with greater emphasis placed upon per cycle foaling rates. Although computer-automated methods have increased repeatability due to the ability of computer algorithms to track swimming speed, agreement between CASA and manual microscopy assessment is frequently reported in several species, including for stallion motility [19]. All subgroups within an individual study, which met the eligibility criteria, were included [24,25].

#### 2.2.2. Article Screening

**Figure 1:**
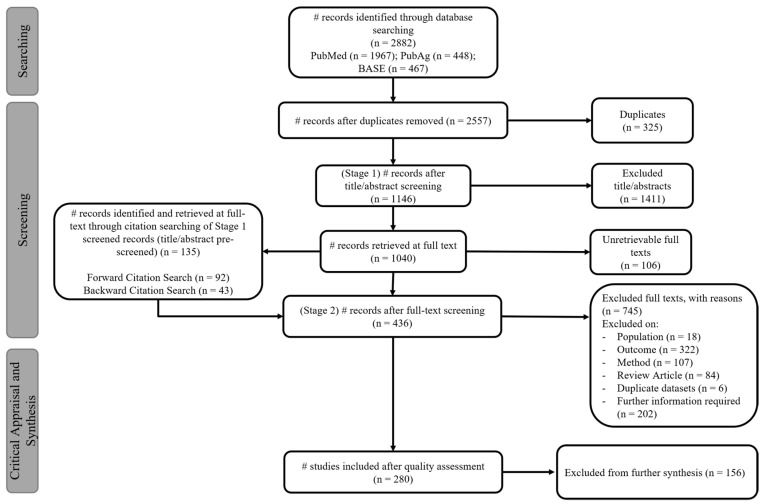
An adapted MOOSE systematic review flow diagram to identify, screen, and critically appraise records for inclusion in the current review [24].

An adapted MOOSE systematic review flow diagram was used to identify and screen articles eligible for inclusion (see Figure 1). Title and abstract screening formed the first stage of the screening process. Studies that indicated stallion PM was a measurable outcome in either the title or abstract were considered eligible. Duplicate datasets were identified and removed at stage one. Unique studies eligible for stage two were exported to NVivo (QSR International version 12) to tabulate justifications for inclusion/exclusion of studies in the stage two screening process. The full text was reviewed and either assigned as eligible for quality assessment or to exclusion with justified reasoning based on pre-defined categories informed by the eligibility criteria [25]. Articles accepted for stage two screening were used to identify additional articles using citation searches. Backward citation searches were conducted using the reference lists of obtained articles [24]. Forward citation searches were conducted by inputting the title of each record into Google Scholar (https://scholar.google.com) and using the “Cited by” function.

### 2.3. Quality Assessment

Assessment of study quality was informed by the Strengthening the Reporting of Observational Studies in Epidemiology (STROBE) Checklist and based on six domains of bias: selection, performance, detection, attrition, reporting, and confounding [25,26]. In response to the signalling questions, a category for high, uncertain, or low risk was assigned for each domain of bias. For each study, an overall judgement was made to accept or reject the study for data extraction based on the risk of bias assigned to each domain [25,26]. Quality assessment was conducted in NVivo to tabulate the risk of bias for each domain [27].

### 2.4. Data Extraction 

Data extraction was performed in MS Excel (Microsoft Office, version 2019). The summary statistics on percentage of the progressively motile sperm (mean, median, standard deviation, standard error) were extracted from 260 peer-reviewed journal articles and 20 grey literature records (Master’s theses, doctoral theses, non-peer reviewed articles, and conference proceedings/abstracts). Additional data on the year of collection, season of collection, geographical location, method of PM assessment (CASA or microscopy), and stallion breed were also extracted (see Table 2). If not specified, the year of sample collection was estimated by calculating the average difference from other publications and subtracting this from year of publication, as followed by other literature [6]. The geographical location was determined by the continent of the first author affiliation and/or the continent in which ethical approval was granted [7]. The breed categories were determined based on the predominant breed-types present among all individual studies [28]. For studies in which the population comprised of multiple breeds, the predominant breed type was chosen for assigning the breed category.

### 2.5. Statistical Analysis 

Data extracted and recorded in the digital spreadsheet were analysed using GraphPad Prism 9 (GraphPad Prism version 9.0, GraphPad Software, California, CA, USA). Statistical analysis was based on 696 unique estimates for PM collected from 280 studies between 1990 and 2018. The Shapiro–Wilk test determined whether parametric or nonparametric statistical tests were used [29]. Simple linear regression was used to predict PM as a function of year of semen collection, and in relation to method of motility analysis (CASA or microscopy), geographical location, and to investigate whether year of semen sample collection predicted PM dependent on preparation method prior to assessment. One-way analysis of variance (ANOVA) or Kruskal–Wallis test investigated the differences in PM between season of collection, geographical location, and breed category. Dunn’s multiple comparison post-hoc test analysed differences between stallion breed and geographical location. A Mann–Whitney test was used to compare significant differences in PM between CASA and microscopy methods. A two-way ANOVA determined whether stallion breed affected PM in raw, fresh, and cool semen samples (Table 1). Tukey’s multiple comparison post-hoc test analysed differences between seasons and stallion breeds, respectively, for PM separated by raw, fresh, and cool semen samples. Results were considered significant when *p* ≤ 0.05.

## 3. Results

The systematic search of stallion semen quality literature returned 2882 records and an additional 135 records through citation searches. Of these, 325 duplicates were removed, 1411 records were excluded after title/abstract screening, 106 could not be retrieved at full text, 436 were excluded after full-text screening, and 156 were excluded from further synthesis after critical appraisal (Figure 1). The results are based on 696 unique estimates for stallion PM from 280 studies between 1990 and 2018.

### 3.1. Year of Semen Collection

**Figure 2:**
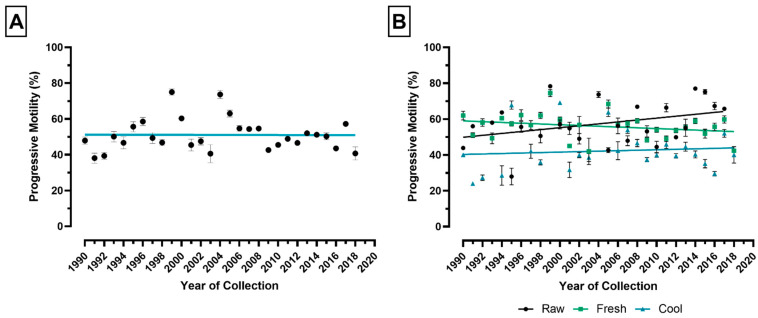
Progressive motility (PM) by year of semen collection in the period from 1990 to 2018. (**A**) PM plotted by year of semen collection. Each point represents the mean (±SEM) PM for a different year. (**B**) PM plotted by year separated by ejaculate type: raw (black), fresh (green), and cool (blue). Each point represents the mean (±SEM) PM for a different year for each ejaculate type. The lines for both figures denotes the best-fit lines. Error bar ± 1 SEM.

The mean percentage of PM in the period from 1990 to 2018 was 51.06% ± 0.33 (±SEM). There was no significant change in PM in the period from 1990 to 2018 (Figure 2A; slope = −0.007497, R^2^ = 0.00005, *p* ≥ 0.05). When PM was separated into raw, fresh, and cooled samples and plotted against the year of semen collection, there was no significant change in PM for all factors (Figure 2B: raw: slope = 0.5337, R^2^ = 0.1326, *p* ≥ 0.05; fresh: slope = 0.0735, R^2^ = 06637, *p* ≥ 0.05; cool: slope = 0.1301, R^2^ = 0.009240, *p* ≥ 0.05). 

### 3.2. Method of Sperm Motility Assessment

**Figure 3:**
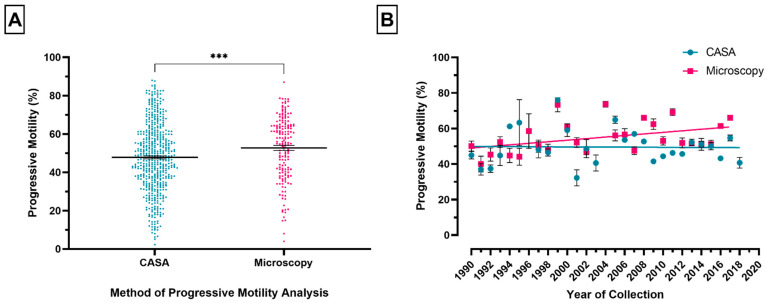
The influence of methods of motility analysis on progressive motility (PM). (**A** )Differences in mean (±SEM) PM between microscopy and CASA. Each point represents the mean PM for each individual study that provided PM estimates assessed by CASA (blue) or microscopy (pink). (**B**) PM plotted by year (1990–2018) separated by motility analysis method: CASA (blue) or microscopy (pink). Each point represents the mean (±SEM) PM for a different year for each method of motility analysis. The lines denote the best-fit lines.*** *p* ≤ 0.001. Error bar ± 1 SEM.

There was a significant difference in PM between the method of motility assessment (*p* ≤ 0.001). Figure 3A shows that PM was significantly higher for studies that assessed motility using microscopy (168 data points) compared to those using CASA (538 data points). There was a significant increase in PM for studies using microscopy in the period from 1990 to 2018, but no significant change for CASA systems overtime (Figure 3B: microscopy: slope = 0.4394, R^2^ = 0.1675, *p* ≤ 0.05; CASA: slope = −0.02396, R^2^ = 0.0004617, *p* ≥ 0.05). 

### 3.3. Breed Category

**Figure 4:**
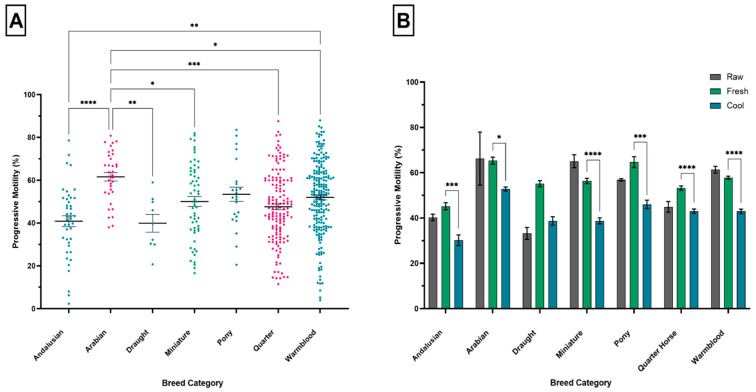
The influence of breed category on progressive motility (PM). (**A**) Differences in mean (±SEM) PM between breed categories. Each point represents the mean PM for individual studies that provided PM estimates for each breed category. (**B**) Effect of ejaculate type on PM within breed categories. Each bar represents the mean (±SEM) PM for raw (grey), fresh (green), and cool (blue) semen samples for each breed category. * *p* ≤ 0.05, ** *p* ≤ 0.01, *** *p* ≤ 0.001, **** *p* ≤ 0.0001. Error bar ± 1 SEM.

There were significant differences in PM between breed categories (*p* ≤ 0.0001; Figure 4A). Figure 4A shows that PM for the Arabian breed category was consistently greater than other breed categories, and significantly greater than Andalusian, Draught, Miniature, Quarter Horse, and Warmblood breed categories (*p* ≤ 0.0001; *p* ≤ 0.01; *p* ≤ 0.05; *p* ≤ 0.0001; *p* ≤ 0.05, respectively). There were significant intra- and inter-breed differences between raw, fresh, and cool semen samples (*p* ≤ 0.0001; *p* ≤ 0.0001; *p* ≤ 0.0001). Figure 4B shows intra-breed differences for PM were consistently lower for cool semen samples compared to fresh semen samples and significantly for lower for all breed categories except Draught (Andalusian fresh:cool *p* ≤ 0.001; Arabian fresh:cool *p* ≤ 0.05; Miniature fresh:cool *p* ≤ 0.0001; Pony fresh:cool *p* ≤ 0.001; Quarter horse fresh:cool *p* ≤ 0.0001; Warmblood fresh:cool *p* ≤ 0.0001). 

### 3.4. Season of Semen Collection

**Figure 5:**
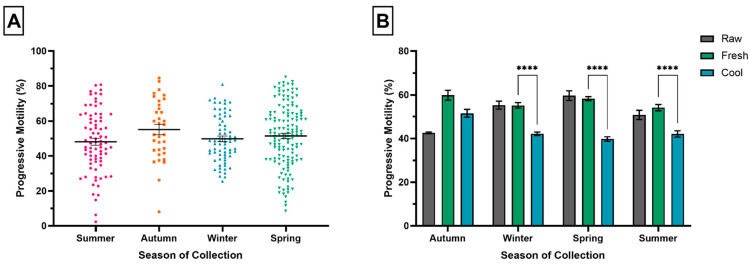
The influence of season of collection on progressive motility (PM). (**A**) Differences in mean (±SEM) PM between seasons of collection. Each point represents the mean PM for individual studies that provided PM estimates for each season. (**B**) Effect of ejaculate type on PM within seasons. Each bar represents the mean (±SEM) PM for raw (grey), fresh (green), and cool (blue) semen samples for each season. **** *p* ≤ 0.0001. Error bar ± 1 SEM.

There was no significant difference in PM between the seasons of collection (*p* ≥ 0.05), with a mean PM of 55.13% ± 2.92, 49.79% ± 1.58, 51.41% ± 1.52, and 48.21% ± 1.96 for autumn, winter, spring, and summer, respectively (Figure 5A). There were significant differences in PM between and within seasons for raw, fresh, and cool semen samples (*p* ≤ 0.0001, *p* ≤ 0.05, *p* ≤ 0.0001). Figure 5A shows PM was consistently lower for cool semen samples compared to fresh, and significantly lower in winter, spring, and summer (*p* ≤ 0.0001, *p* ≤ 0.0001, *p* ≤ 0.0001). For cool semen samples, PM was significantly higher in the autumn compared to winter, spring, and summer (*p* ≤ 0.05, *p* ≤ 0.0001, *p* ≤ 0.01). 

### 3.5. Geographical Location

**Figure 6:**
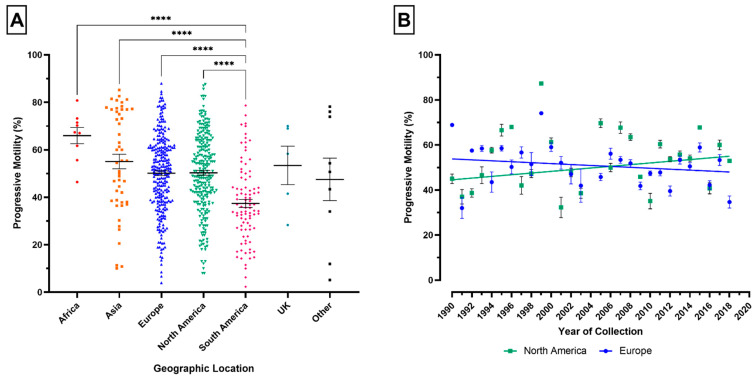
The influence of geographical location on progressive motility (PM). (**A**) Differences in mean (±SEM) PM between geographical locations. Each point represents the mean PM for individual studies that provided PM estimates for each geographical location. (**B**) PM plotted by year (1990–2018) separated by geographical location: North America (green) and Europe (blue). Each point represents the mean (±SEM) PM for a different year for North America and Europe. The lines denote the best-fit lines. **** *p* ≤ 0.0001. Error bar ± 1 SEM.

There were significant differences in PM between geographical locations (*p* ≤ 0.0001). Figure 6A depicts that PM was consistently lower for stallions located in South America, and significantly reduced compared to Africa, Asia, Europe, and North America (*p* ≤ 0.0001, *p* ≤ 0.0001, *p* ≤ 0.0001, and *p* ≤ 0.0001). There were significant changes in PM over time for both Europe and North America (*p* ≤ 0.05, *p* ≤ 0.0001). Figure 6B shows PM significantly decreased in Europe (slope = −0.2073, R^2^ = 0.01709) and significantly increased in North America (slope = 0.3737, R^2^ = 0.06986) in the period 1990–2018.

## 4. Discussion

Our findings represent the status of equine PM across the last three decades, for which research is limited [16]. This present study suggests the fertilising capacity of equine ejaculate has remained consistent, but there is considerable variation in PM.

Variations in stallion PM have been reported to account for only 20% of the total variation in fertility, as such, PM below 40% is likely to compromise stallion fertility [30]. A study utilising equine embryo recovery rates identified that a PM greater than or equal to 45% is the threshold value associated with a change from lesser to higher fertility [31]. Our findings highlight that stallions with no history of perturbed reproductive health have a current PM globally that is above the threshold for high fertility [30,31]. Despite significant seasonal differences between fresh and cool ejaculates, overall seasonal differences were not significant and ejaculate type did not significantly change PM overtime. Since PM is considered an important parameter for fecundity, our results suggest satisfactory reproductive performance can be achieved outside optimal breeding periods, irrespective of whether ejaculates are fresh or cool-stored. Inferences drawn from retrospective analysis are limited due to inherent heterogeneity between individual studies, but our study is the first to examine temporal trends in stallion PM and provides avenues for future stallion fertility research.

It is argued that changes in human laboratory andrology methodologies resulted in systematic errors being interpreted as a decline in semen quality [3,10]. Data presented here show a significant temporal increase in equine PM for studies utilising microscopy, but no significant change in PM over time for studies utilising CASA. The findings presented here highlight the extent to which methodological differences, incorporating visual assessment, contribute to variations in equine PM, manifested by the significant modification of temporal trends. 

Graphically, equine PM appears to be normally distributed for studies using CASA, while microscopy has a distinct cluster of studies at a greater motility [29]. Within the literature, boar ejaculates assessed visually reported significantly higher PM (77%) compared to CASA (39%), consistent with our results [32]. The results of the present study propose CASA to be a more consistent methodology for equine motility assessment and underscore the considerable heterogeneity of microscopy studies due to technical variability [10,25,33]. Evaluation of the canine ejaculate by different technicians has demonstrated high inter-observer variability (30–60%), highlighting that the skills, experience, and training of individual technicians can influence the interpretation of motility [34]. Methodological differences within and between laboratories over time may explain the considerable variation in PM. Evidence of individual stallion and inter-breed variation indicates methodological changes overtime are not solely responsible [35,36,37,38].

The significance of breed variations in equine PM is evident, since semen traits have been found to be related to genotype and breed, and are therefore heritable [37,38]. This study provides further evidence that breed could account for the considerable variation in semen quality among stallions, manifested by significant between-breed variation in PM. Breed variations in equine PM reported here are consistent with literature reporting whereby Arabian stallions exhibit greater PM compared to warmblood and light horse breeds [28,39,40]. In mares, reports suggest reproductive genes involved in oocyte maturation, development, and function differ amongst Spanish breeds alongside certain fertility traits [41,42]. Evidence of breed differences in reproductive traits indicates significant differences in equine PM may be a function of genetic factors. Methodological and technical differences between individual studies limits assumptions on the extent breed differences contributed to variations in PM [10,25]. 

In human reproductive studies, geographical location is a potential confounder for variations in semen quality. Our study included global stallion PM data, as such geographic location was varied and unequally distributed. Therefore, variations should be interpreted with caution. Europe and North America provided similarly distributed datasets to further investigate geographical differences from a temporal aspect. Included studies from Europe assessing stallion PM parallel the declines observed in European human semen quality and for UK stud dogs [5,12,43]. The increase in PM for stallions in North America conflicts with existing human semen quality data, perhaps related to inherent limitations of meta-analyses or an effect of inter-species differences [3,6,44]. To interpret geographical differences in stallion PM, the method of motility analysis could be considered a confounding factor in the present study due to the inclusion of both manual and computer-automated methods within this meta-analysis. Steeper declines in human semen quality and higher incidences of testicular cancer have been observed in Europe compared to other continents [7,45,46]. Taken with the data presented here, it could be inferred that an environmental aetiology may be at least partly responsible for geographical differences in equine PM, presenting an area worthy of future consideration.

Environmental pollution and contaminants are reported for impairing sperm quality and causing reproductive abnormalities [47]. The relationship between geographical variations in semen quality and the potential burden of environmental pollution is contentious, yet sufficient observational and experimental data exist to indicate there is a cause for concern. Research concerning exposure levels of environmental contaminants in the equine is limited; however, environmental pollutants, namely polychlorinated biphenyls, have been detected in horse meat and donkey milk [48,49]. In Pennsylvania (USA), there were increased incidences of dysphagic foals born in an active unconventional gas development, highlighting areas within North America with high environmental chemical burdens [50]. For Europe, a higher proportion of studies of particularly low progressive motility may have originated from contaminated areas that may have confounded the progression lines in the current meta-analysis, similarly for North America. Single-centre studies would provide a more accurate representation of semen quality trends with the potential to identify sources of variation across regions.

The prognostic value of our results as markers for stallion fertility potential cannot be substantiated due to the lack of standardised reference limits for semen parameters in the equine sector [19]. For human sperm quality analysis, the WHO guidelines that define PM when using manual and computer-automated methods are the same, and these give rise to the recommended categories and sperm swimming speeds for progressive motility assessment. This information can be placed into CASA algorithms to assess sperm motility. Information on the direct CASA algorithms for sperm swimming speed in data obtained for this study was not possible. Development of internationally accepted equine sperm quality reference limits would increase consistency across the equine sector in stallion semen evaluations for the selection of future breeding prospects [51]. Using equine pregnancy rates as comparative markers for stallion sperm quality reference limits, similarly to the WHO reference limits for human sperm quality, the predictive value of semen parameters to assess stallion fertility potential could be improved [1,52]. Determining the source of variation in equine PM is challenging due to the inherent limitations of heterogeneity in meta-analytical studies. Missing data for variables of interest limited the choice of statistical methods. The statistical methods used limit equine PM inferences as they did not account for within and between study variation. Variations in equine PM could be related to discrepancies within the data at breed-level. Grouping breeds is easier when genetic lines are similar, as seen in the livestock industry in which genetic differences arise due to different breeding goals [53]. Genetic lines within dairy cow breeds are similar but are genetically different to beef cattle or dual-purpose breeds [53]. This inconsistency is representative of some of the wider challenges of heterogeneity in retrospective analysis, particularly within the equine sector. There is a need for validation and standardisation of objective methodologies to assess equine motility to replace visual estimation to reduce technical variability within and between laboratories [10,33,51]. An international collaboration utilising standardised objective motility analysis would overcome inherent limitations in methodological differences in retrospective analysis and provide a more robust explanation for variations in equine PM. 

## 5. Conclusions

Monitoring stallion reproductive trends can inform decisions on the selection of future breeding prospects to navigate the potential economic impacts of poor reproductive efficiency. The results from the present study indicate stallion PM has remained unchanged globally across the last three decades. Comparative markers to assess the fertility potential of equine sperm values would increase the predictive value of our results. Significant differences between methods of motility assessment showcase the wide variability in semen evaluation in the equine sector. The present study highlights the need for standardisation across the equine breeding industry to support stallion fertility research. Research should focus on validating objective methodologies to assess equine motility to promote consistency across the equine sector. It is the authors’ recommendation that implementation of internationally-accepted semen evaluation methodologies be developed to reduce technical variability, allowing for geographical and breed-level variations to be further explored.

## Figures and Tables

**Table 1:** Eligibility criteria for the systematic review and meta-analysis.

Inclusion	Exclusion
Domesticated *Equus caballus* only. Semen collection via an artificial vagina including Hannover, Colorado, Missouri, French, Botupharma, Avenches, and Roanoke models. Semen collection utilising a phantom, live mare, or via ground collection. Semen quality analysis of progressive motility. Progressive motility assessment via Computer-Assisted Sperm Analysis or microscopy analysis. Semen samples assessed less than 24 h after collection without the addition of extender (raw), or with the addition of an extended (fresh). Semen samples assessed after cool-storage within a 72-h period (cool).	Alternative sub-species of the *Equus* genus. Non-standard methods of semen collection, including epididymal retrieval or stimulation of ejaculation via pharmacological methods or electroejaculation. Semen quality parameters given for sexed semen samples. Stallions displaying signs of perturbed reproductive health including anatomical, seminal, and bacterial or poor libido. Parameters recorded for cryopreserved or thawed semen samples.
English language documents only. Peer-reviewed published literature including primary and retrospective data sets and case reports. Academic grey literature including dissertations and theses, conference presentations, and posters.	Published in a language other than English and without translation. Data presented in the format of a review article or opinion article. Duplicate datasets.

**Table 2:** Categorisation of variables for statistical analysis (geographic location, breed, and season of collection) based on data extracted from individual studies.

Category for Analysis	Data Extracted from Individual Studies	
**Geographic Location**	**Countries Included**	
Africa	Egypt, South Africa	
Asia	India, Iran, Saudi Arabia, Thailand	
North America	United States, Canada, Mexico	
South America	Argentina, Brazil, Chile, Colombia	
Europe	Austria, Belgium, Czech Republic, Finland, France, Germany, Italy, Netherlands, Poland, Portugal, Spain, Sweden, Switzerland	
UK	United Kingdom	
Other	Australia, New Zealand, Russia, Turkey	
**Breed category ^1^**	**Horse breeds included**	
Andalusian	Lusitano, Peruvian Paso, Lipizzaner, Mangalarga Marchador, Brazilian Jumping, Spanish Purebred, Sorraia, Garrano	
Arabian	Arabian, Anglo-Arab	
Draught	Draught, Polish Coldblood	
Miniature	Miniature, Shetland, Miniature Caspian Pony	
Pony	Brazilian Pony, Pony, Connemara, Welsh Pony	
Quarter Horse	American Quarter Horse; Azteca Horse, Thoroughbred; Trakehner, Manipuri, Standardbred, American Paint Horse, Friesians, Thai Native X, Pantaneiro, Trotter, Maremmano, Finnhorse	
Warmblood	Hanoverian, Holsteiner, Appaloosa, Dutch Warmblood, German Warmblood, Oldenburg Warmblood, Rhinelander, Chilean Purebred, Marwari, Kathiawari, Zanskari, French Saddlebred, Westphalian, Swedish Warmblood, Old Kladruber, Criollo Colombiano, Brandenburg, Belgium Draft, Franches-Montagnes, French Warmblood, Polish Warmblood, Haflinger	
**Month of Collection**	**Northern Hemisphere**	**Southern Hemisphere**
November-February	Winter	Summer
March-June	Spring	Autumn
July-August	Summer	Winter
September-October	Autumn	Spring

^1^ Breed category = breed categories were determined based on the predominant breed types reported among individual studies. For studies in which the population comprised of multiple breed types, the predominant breed type was chosen for assigning the breed category, as such breed categories are not exclusive.

## Data Availability

No new data were created or analysed in this study. Data sharing is not applicable to this article.
